# Ferroptosis, pyroptosis and necroptosis in acute respiratory distress syndrome

**DOI:** 10.1038/s41420-023-01369-2

**Published:** 2023-03-10

**Authors:** Yongxin Zheng, Yongbo Huang, Yonghao Xu, Ling Sang, Xiaoqing Liu, Yimin Li

**Affiliations:** Department of Respiratory and Critical Care Medicine, the First Affiliated Hospital of Guangzhou Medical University, Guangzhou Institute of Respiratory Health, State Key Laboratory of Respiratory Diseases, 510120 Guangzhou, China

**Keywords:** Respiratory distress syndrome, Cell death

## Abstract

Acute respiratory distress syndrome (ARDS) is an acute and uncontrolled pulmonary inflammation caused by various insults. Cell death is a critical mechanism in the pathogenesis of ARDS. Ferroptosis, a novel form of cell death defined as iron-mediated lipid peroxidation, has been shown to play a role in the pathogenesis of ARDS. Additionally, pyroptosis and necroptosis are also involved in the pathophysiological process of ARDS. The crosstalk among ferroptosis, pyroptosis, and necroptosis is getting increasing attention. Therefore, this review will mainly summarize the molecular mechanisms and central pathophysiological role of ferroptosis in ARDS. We will also discuss our understanding of pyroptosis and necroptosis as they pertain to the pathogenesis of ARDS. Furthermore, we also describe the pathological processes that engage crosstalk among ferroptosis, pyroptosis, and necroptosis. We consider that individual pathways of ferroptosis, pyroptosis, and necroptosis are highly interconnected and can compensate for one another to promote cell death.

## Facts

- Ferroptosis is a unique type of cell death characterized by iron-dependent lipid accumulation.
- Ferroptosis, pyroptosis, and necroptosis are immunogenic forms of cell death, which are associated with uncontrolled inflammatory damage.
- There is interconnectivity among ferroptosis, pyroptosis, and necroptosis.
- Targeting ferroptosis holds great potential in treating ARDS.

## Open questions

- How does the iron-dependent lipid metabolism execute and distinguish ferroptosis from pyroptosis and necroptosis?
- What is the specific role of ferroptosis, pyroptosis, and necroptosis in ARDS?
- What is the cross-regulation among ferroptosis, pyroptosis, and necroptosis in ARDS?
- How can we target ferroptosis as a potential treatment for ARDS?

## Introduction

Acute respiratory distress syndrome (ARDS) was first defined by Ashbaugh in 1967 in a case-series report [1]. According to the Berlin definition [2], ARDS is a life-threatening condition that can be caused by both pulmonary (e.g., pneumonia and pulmonary embolism) and nonpulmonary (e.g., sepsis and trauma) insults, leading to hypoxemia, non-hydrostatic pulmonary edema and pulmonary extracellular matrix (ECM) remodeling [2, 3]. ARDS, with high morbidity and mortality rate, accounts for 10% of intensive care unit admissions and affects more than 3,000,000 patients annually worldwide. Besides, according to the US report, there are more than 200,000 cases and 75,000 deaths in the US annually [4]. The pathogenesis of ARDS is believed to involve the accumulation of inflammatory cells, oxidative stress, cell death, and so on [3, 5, 6].

Ferroptosis is a unique form of cell death that was first discovered in 2012 [7]. Unlike apoptosis, autophagy, pyroptosis, and necroptosis, ferroptosis is characterized by the overwhelming, iron-dependent accumulation of lethal lipid reactive oxygen species (ROS) [7]. Numerous studies have demonstrated that ferroptosis plays an important role in ARDS. The ferroptosis inhibitors (e.g., ferrostatin-1 (Fer-1) and lipoxstatin-1) can significantly ameliorate ARDS by decreasing inflammation and oxidative stress by inhibiting ferroptosis [8, 9]. Thus, in this review, we will focus on the molecular regulatory mechanisms of ferroptosis in ARDS, as well as the association of pyroptosis, necroptosis, and ferroptosis with the pathogenesis of ARDS.

## Ferroptosis, a unique form of cell death

Ferroptosis is remarkably distinct from other forms of cell death in terms of morphological, biochemical, and genetic features (Table 1). It is a peroxidation-mediated form of programmed cell death (PCD) that requires abundant and accessible cellular iron. It was unknown until the discovery of ferroptosis inducers [10]. Erastin and RSL3, the first ferroptosis inducers, were discovered by high-throughput screening of small molecule libraries [11, 12]. Erastin and RSL3 treatment triggered cell death with distinct morphological changes and biochemical processes which could not be attenuated or reversed by caspases inhibition, necroptosis inhibitors (e.g., necrostatin-1), or pharmacological inhibition of autophagy (e.g., chloroquine and 3-methyladenine) [13]. However, the cell death induced by Erastin and RSL3 can be reversed by the iron chelators (e.g., deferoxamine mesylate) or antioxidants (e.g., ferrostatin-1 and glutathione (GSH)) which strongly suppress the lipid ROS generation [14–16]. Thus, the type of cell death induced by Erastin and RSL3 may be proposed to be an iron-dependent, ROS-accumulation form of PCD named ferroptosis [7].

**Table 1:** A comparison of features associated with various types of programmed cell death [16, 121–125].

Type	Morphological features	Biochemical features	Immune features	Positive regulators	Negative regulators
Ferroptosis	Cell membrane: plasma membrane blebbing and lacking rupture Cytoplasm: shrunken mitochondria, increased mitochondrial membrane density, disruption of membrane integrity. Nucleus: lack of chromatin condensation and margination.	Iron and ROS accumulation. Formation of lipid peroxidation products (e.g., MDA and 4-HNE). GSH depletion. NADPH oxidases (NOXs) are activated and released by the arachidonic acid mediators.	Proinflammatory due to the release of DAMPs. Activation of NF-κB and MAPK pathways	VDAC2/3 NCOA4 NOXs ALOXs P53 TFR1 FTH1 ACSL4 PTGS2	GPX4 SLC7A11 GSH NRF2 HSPB1
Pyroptosis	Cell membrane: cell swelling and plasma membrane blebbing. Cytoplasm: formation of vesicles and inflammasomes. Nucleus: chromatin condensation and nuclear fragmentation.	Activation of caspases 1/4/5/11 and GSDMD cleavage. Releasing IL-1β and IL-18.	Robust proinflammatory due to release inflammatory factors and DAMPs.	Caspases 1/3/4/5/8 PRKN GSDMD IRGB10 TLR7	
Necroptosis	Cell membrane: cell shrinkage and plasma membrane blebbing. Cytoplasm: cytoplasmic and cytoplasmic organelles swelling. Nucleus: moderate chromatin condensation.	Activation of RIPK1, RIPK3, and MLKL and formation of necrosome. Drop in ATP. Releasing DAMPs.	Most often proinflammtory due to the release DAMPs. In some cases anti-inflammatory	RIPK1, RIPK3 MLKL TNFR1	STUB1 A20 AURKA Protein phosphatase
Apoptosis	Cell membrane: plasma membrane blebbing and cell shrinkage. Cytoplasm: cleavage of cytoskeletal proteins and collapse of subcellular components. Nucleus: chromatin condensation and nuclear fragmentation.	Caspases activation and cleave numerous proteins. Fragmentation of DNA.	Often anti-inflammatory and immune silent. In some cases proinflammatory	P53 Caspases Bax Bak Fas FasL	Bcl-2 Bcl-XL
Autophagy	Cell membrane: lack of change and may exist the plasma membrane blebbing. Cytoplasm: swelling of cytoplasmic organelles and formation of autophagosomes. Nucleus: nuclear fragmentation and lack of chromatin condensation.	LC3-I to LC3-II conversion Substrate (e.g., p62) degradation.	Most often anti-inflammatory due to inhibit the inflammasome activation. Proinflammatory due to mediation of secretion of cytokines.	ATG5 ATG7 ATG3 Utx Beclin 1 Rala	

*VDAC* voltage-dependent anion channel, *NCOA4* nuclear receptor coactivator 4, *ALOXs* arachidonate lipoxygenase, *TFR1* transferrin receptor 1, *FTH1* ferritin heavy polypeptide 1, *ACSL4* acyl-CoA synthetase long-chain family member 4 acyl-CoA synthetase long-chain family member 4, *PTGS2* prostaglandin-endoperoxide synthase 2, *HSPB1* heat shock protein family B (small) member 1, *GPX4* glutathione peroxidase 4, *SLC7A11* solute carrier family 7 member 11, *GSH* glutathione, *NRF2* nuclear factor erythroid 2-related factor 2, *PRKN* parkin RBR E3 ubiquitin protein ligase, *GSDMD* gasdermin-D, *IRGB10* immunity-related GTPase B10, *TLR* toll-like receptor, *RIPK* receptor interacting protein kinases, *MLKL* mixed-lineage kinase domain-like protein, *TNFR1* tumor necrosis factor receptor-1, *STUB1* STIP1 homology and U-Box containing protein 1, *AURKA* aurora kinase A, *ATG* autophagy related, *Utx* ubiquitously transcribed tetratricopeptide repeat on chromosome X, *Rala* RAS like Proto-Oncogene A, *MDA* malondialdehyde, *4-HNE* 4-hydroxynonenal.

## Mechanisms of ferroptosis

**Fig. 1:**
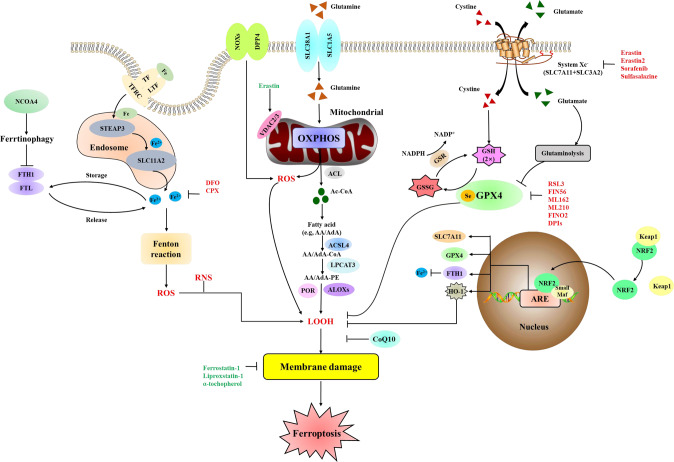
Molecular mechanism and signaling pathways of ferroptosis. Ferroptosis is driven primarily through two major pathways. The extrinsic or transporter-dependent pathway (e.g., increased iron uptake and decreased cysteine or glutamine uptake), and the intrinsic or enzyme-regulated pathway (e.g., the inhibition of GPX4 or activation of ALOXs in the lipid metabolic pathway). TF transferrin, LTF lactotransferrin, TFRC transferrin receptor, SLC11A2 solute carrier family 11 member 2, FTL ferritin light chain, RNS reactive nitrogen species, DPP4 dipeptidyl peptidase 4, SLC38A1 solute carrier family 38 member 1, SLC1A5 solute carrier family 1 member 5, OXPHOS oxidative phosphorylation, ACL ATP citrate lyase, Ac-CoA acetyl-CoA, AA arachidonic acid, AdA adrenic acid, CoA coenzyme A, ACSL4 acyl-CoA synthetase long-chain family member 4, PE phosphatidylethanolamine, LPCAT3 lysophosphatidylcholine acyltransferase 3, POR cytochrome p450 oxidoreductase, LOOH lipid hydroperoxides, SLC3A2 solute carrier family 3 member 2, GSSG GSH disulfide, GSR glutathione-disulfide reductase, ARE antioxidant response element, HO-1 heme oxygenase-1, DFO deferoxamine, CPX ciclopirox olamine, DPIs diphenyleneiodonium chloride.

Ferroptosis is regulated by lipid peroxidation and iron accumulation. Thus, the increased free radical production, fatty acids supply, and lipid peroxidation by dedicated enzymes is critical for ferroptosis (Fig. 1) [17]. Besides, the antioxidant system, including enzymatic and non-enzymatic antioxidants, can stabilize or scavenge free radicals to inhibit ferroptosis.

## Oxidant systems

### Production of free radicals

**Fig. 2:**
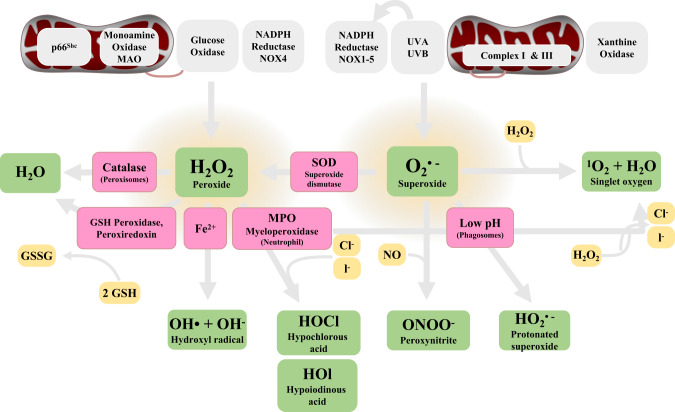
The mechanisms and pathways for generating common ROS products. The NADPH oxidases (NOXs) family, Xanthine oxidase, Myeloperoxidase (MPO), and GSH peroxidase are important enzymes that promote ROS production in the cells. Activation of these pathways combined with the abnormal antioxidant mechanisms (e.g., superoxide dismutase (SOD)) will result in oxidative stress and ferroptosis.

Free radicals, including ROS and reactive nitrogen species (RNS), are oxidants produced by redox reactions, which participate in the oxidation of cellular components and regulate cell death. Compared to the RNS, the functions, and mechanisms of ROS in ferroptosis have been well studied. ROS are byproducts of mitochondrial metabolism and include hydrogen peroxide (H_2_O_2_), hydroxyl radicals (OH•), superoxide anion (O_2_^•–^), and singlet oxygen (^1^O_2_) [18, 19]. ROS can interconvert from one to another by enzymatic and non-enzymatic mechanisms (Fig. 2). ROS are produced by iron-mediated Fenton reaction, NADPH oxidases (NOXs) family, and oxidative phosphorylation (OXPHOS) in mitochondrial. In the electron transport pathway, ROS can be converted to hydrogen peroxide (H_2_O_2_) by superoxide dismutase (SOD). Then, cells with high level ferric iron (Fe^2+^) will initiate the Fenton reaction which will further promote the conversion of H_2_O_2_ to high toxic hydroxyl radicals (OH• and O_2_^•–^) [20]. The NOXs family participates in a membrane-bound enzyme complex that can transport electrons across the plasma membrane to yield superoxide and other downstream ROS [21, 22]. The production of ROS can promote lipid peroxidation to induce ferroptosis. Additionally, mitochondrial metabolism is also an important source of ROS [23]. An imbalance of the generation and clearance of ROS leads to oxidative stress, which can damage DNA, proteins, and lipids [24]. A full understanding of the ROS regulatory network will help us understand the mechanisms of ferroptosis. However, there is a need to further clarify the interactions and differences among different ROS sources in the regulation of ferroptosis.

### Fatty acids supply

Fatty acids, including polyunsaturated fatty acids (PFUA) and monounsaturated fatty acids (MUFAs), have been shown to play an important role in ferroptosis. Dierge et al. [25] have demonstrated that an excess uptake of PFUAs could lead to ferroptosis of tumor cells, which exhibits an anti-tumor effect. On the other hand, exogenous MUFAs can inhibit ferroptosis by ameliorating the lipid ROS accumulation at the plasma membrane and decreasing levels of phospholipids containing oxidizable PFUAs [26]. In cells, ROS can react with PFUAs of lipid membranes to induce ferroptosis. Lysophosphatidylcholine acyl-transferase 3 (LPCAT3) [27] and acyl-CoA synthetase long-chain family member 4 (ACSL4) [28] have been identified as the critical enzymes to promote the production of PFUA derivatives. Thus, suppression of LPCAT3 and ACSL4 will decrease the oxidative PFUAs in the membrane and inhibit ferroptosis (Fig. 1). Besides, AMP-activated protein kinase (AMPK), as a sensor of cellular energy status, also regulates the ferroptosis by mediating phosphorylation of acetyl-CoA carboxylase (ACC) and PFUAs biosynthesis [29]. However, AMPK also mediates the phosphorylation of beclin 1 to promote ferroptosis [30]. Thus, further studies are needed to clarify the mechanisms of AMPK in regulating ferroptosis.

### Lipid peroxidation

Lipid peroxidation is induced by both enzymatic and non-enzymatic means. The mammalian arachidonate lipoxygenase (ALOX) family, consisting of six members (ALOXE3, ALOX5, ALOX12, ALOX12B, ALOX15, and ALOX15B) [31], can mediate PUFA peroxidation to produce initial lipid hydroperoxides (LOOH) and subsequent reactive aldehydes, such as malondialdehyde (MDA) and 4-hydroxynonenal (4-HNE). These aldehydes not only damage the DNA and protein but also promote and amplify inflammation [17]. Thus, depletion of ALOXs in cells can prevent ferroptosis induced by Erastin and ameliorate inflammation [32, 33]. Despite the important role of ALOXs in regulating ferroptosis, other non-ALOXs may also be involved in lipid peroxidation. Cytochrome P450 oxidoreductase (POR), a non-ALOXs pathway, can promote PUFA peroxidation by directly supplying electrons to the P450 enzyme (Fig. 1). The two cofactors, flavin mononucleotide (FMN) and flavin adenine dinucleotide (FAD), combine with POR which can promote auto-oxidation of PUFA to generate ROS [34, 35]. Additionally, prostaglandin-endoperoxide synthase 2 (PTGS2/COX2) also had been considered as a biomarker, not a driver of ferroptosis, which contributes to ferroptosis by indirectly oxidizing lysophospholipids [36–38]. However, it is still unclear whether these oxidases have a similar role in regulating ferroptosis. More research is necessary to understand the role of different oxidases in mediating ferroptosis.

## Antioxidant systems

### System Xc^−^ and GPX4

System Xc^−^ is an important antioxidant system that inhibits ferroptosis (Fig. 1). It is composed of two core subunits: the light-chain subunit SLC7A11 and the heavy-chain subunit SLC3A2. System Xc^−^ is an amino acid transporter that imports cystine and exports glutamate in a 1:1 ratio. The imported cystine can be used in the synthesis of GSH. The inhibition of SLC7A11 by small-molecule compounds (e.g., Erastin) or drugs (e.g., Sorafenib and Sulfasalazine) or glutamate will cause GSH depletion to trigger ferroptosis [7, 39]. GSH can regulate the activity of glutathione peroxidase 4 (GPX4) as a powerful antioxidant. GPX4, the key regulator of ferroptosis, can reduce phospholipid hydroperoxide production by reducing GSH to oxidized glutathione (Fig. 1) [15, 36]. Selenium is required by GPX4 to prevent ferroptosis. Active selenol is oxidized by peroxide to selenic acid and finally reduced by GSH to glutathione disulfide (GS-SG) [15, 40]. Some small molecule compounds (e.g., RSL3, ML162, ML210, FIN56, and FINO_2_) can directly or indirectly induce ferroptosis by inhibiting the activity of GPX4, whereas overexpression of GPX4 can lead to the resistance of ferroptosis [36, 41–44]. Interestingly, emerging studies have revealed that GPX4 can cause other types of cell death, including apoptosis [45], autophagy [46], necroptosis [47], and pyroptosis [48] which means that there is potential interconnectivity between ferroptosis and other PCD. Therefore, it is important to further explore the potential link between cell death and GPX4 in the process of diseases. Moreover, the regulatory mechanisms among the different types of cell deaths warrant further exploration.

### NRF2

The transcription factor Nuclear factor E2 related factor 2 (NRF2) and its negative regulator, kelch-like ECH-associated protein 1 (Keap1), are critical to defend the oxidative stress and maintain the oxidative steady state (Fig. 1). In mammalian cells, Keap1 functions as the molecular sensor for reactive species. Under basal conditions, Keap1 readily binds with NRF2 and tethers it for ubiquitination and proteasomal, thereby maintaining a low level of NRF2. In the intense oxidative stress response, Keap1 is modified on some specific cysteine moieties which disable its E3 ligase adaptor activity. As the result, NRF2 is stabilized and increased via de novo protein synthesis. When the increased abundance of NRF2 exceeds the Keap1 abundance, NRF2 can be activated by detaching from Keap1 and translocated to the nucleus. In the nucleus, NRF2 will increase transcription of the genes that encode antioxidant proteins (e.g., heme oxygenase-1 (HO-1) and ferritin heavy chain 1 (FTH1)) by binding to the antioxidant response element (ARE) [49]. NRF2 plays a critical role in regulating lipid peroxidation and iron/heme metabolism [50–52]. Genetic or pharmacologic inhibition of NRF2 expression or activity in hepatocellular carcinoma cells (HCC) can promote Erastin or Sorafenib-induced ferroptosis, thereby increasing the antitumor effects [53]. Besides, studies have shown that NRF2/HO-1 pathway can regulate ferroptosis and improve inflammation. The NRF2/HO-1 pathway interacts with SLC7A11 to dramatically attenuate ferroptosis [54]. However, some studies have pointed out that the expression of HO-1 can be upregulated by increased heme, which promotes Erastin-induced ferroptosis, suggesting HO-1 has a dual role in ferroptotic cell death [55].

## Programmed cell death in ARDS

### Ferroptosis in ARDS

ARDS is a fatal clinical syndrome characterized by uncontrolled and self-amplifying pulmonary inflammation. ARDS involves damage to alveolar epithelial and endothelial barriers, leading to protein and liquid leakage into the alveoli and interstitium [3, 6]. Oxidative stress has been demonstrated to be associated with the pathogenesis of ARDS. Pathogens can induce the production of ROS to damage the balance between oxidative and antioxidant capacity, resulting in redox imbalance of the local microenvironment and induction of ferroptosis [56].

Ferroptosis can result in the accumulation of immune cells and promote the release of proinflammatory cytokines, which can aggravate lung injury. Thus, ferroptosis is considered as an immunogenic form of cell death [57, 58]. Moreover, inflammation can further promote ferroptosis. Treatment cells with tumor necrosis factor (TNF)-α have been shown to suppress the expression of GPX4 and further promote ferroptosis [59]. Therefore, ferroptosis and inflammation form a self-amplified loop, which further promotes organ damage. Numerous studies have demonstrated that ferroptosis plays an important role in various pathophysiologic models of ARDS. Inhibiting the SLCA11/GPX4/GSH signaling pathway can induce the accumulation of ROS in lung epithelial cells, which causes ferroptosis, as well as the impaired epithelial–endothelial barrier. In sepsis-induced ARDS, treatment of lipopolysaccharide (LPS) can significantly suppress the expression level of GPX4 and SLC7A11, while the level of MDA, 4-HNE, and total iron is strikingly increased. Inhibiting ferroptosis by Fer-1 can reverse these changes and improve inflammatory lung injury [8]. Besides, intestinal ischemia/reperfusion enhanced ferroptosis in lung epithelial cells by inhibiting GPX4, which contributes to the development of ALI [54]. Acute radiation-induced lung injury (RILI) [60] and oleic acid-induced ALI models [61] have also been proven that high levels of ROS-induced oxidative damage to lung tissue. Ferroptosis is a key factor in promoting the development of ALI. However, few studies have been conducted to investigate the potential role of ferroptosis in the pathogenesis of virus-induced ARDS (e.g., influenza and SARS-CoV-2-associated ARDS). Thus, it is urgent to explore the roles and mechanisms of ferroptosis in virus-induced ARDS.

Inhibiting ferroptosis has been demonstrated as an effective approach to alleviate pulmonary inflammation and tissue damage in ARDS. NRF2-mediated antioxidant pathway activation can maintain cellular redox homeostasis and reduces oxidative damage [49, 56]. Recent studies have shown that NRF2 could increase the expression level of HO-1 and SLC7A11 and dramatically attenuate ferroptosis in the ischemia/reperfusion-induced ALI (IIR-ALI) model [54]. Furthermore, NRF2 increases the expression of SLC7A11 by regulating STAT3, indicating that NRF2 can inhibit ferroptosis by regulating inflammation [62]. Thus, more work is warranted to fully understand the crosstalk of NRF2 and STAT3 on ferroptosis. Additionally, iASPP, an inhibitor of p53 which is an apoptosis-stimulating protein, could inhibit ferroptosis and provide protection against ALI via the NRF2/HIF-1/TF-signaling pathway. The levels of inflammatory cytokines (TNF-α, interleukin (IL)-6, and IL-1β) were also dramatically reduced by inhibiting ferroptosis [9]. Thus, NRF2 may be a promising therapeutic target for ARDS/ALI. The mechanisms of NRF2 in regulating ferroptosis should be further explored and clarified. Additionally, NRF2 regulates iron metabolism to decrease oxidative stress. Disruption of iron homeostasis and accumulation of iron can cause oxidative stress and tissue damage through ferroptosis [63]. NRF2 could regulate heme-bound iron and a labile iron pool to synthesize heme or iron–sulfur clusters. Activated NRF2 could reduce cytosolic labile iron, restore homeostasis in situations of cellular iron overload and prevent oxidative stress [50]. Thus, NRF2 is expected to protect cells against ferroptosis by regulating iron. The degradation of target genes FTL and FTH1 could promote ferroptosis. However, it remains to be uncovered how NRF2-mediated ferroptosis protects ARDS by regulating iron and how those pathways converge with iron proteins such as FTL or FTH1. In addition to the NRF2 pathway, recent studies have proven that activating the α7 nicotinic acetylcholine receptor (α7nAchR) in lung tissue [64] or blocking mTOR signaling [65] can significantly ameliorate the sepsis-induced ARDS by inhibiting ferroptosis. The expression of GPX4 and SLC7A11 increased and the iron level decreased. Therefore, there may be many undiscovered pathways and mechanisms related to the development of ferroptosis. Future work is needed to explore and clarify the interactions between these pathways and mechanisms in the regulation of ferroptosis.

Besides, ferroptosis, as immunogenic cell death, has been demonstrated to regulate the immune response in various diseases, particularly cancer. Currently, the role of ferroptosis in tumor suppression by the immune system has been extensively studied [66]. Immune checkpoint blockade therapy by activating T cells is a highly effective class of anti-cancer therapy. Wang et al. reported that ferroptosis-specific lipid peroxidation in tumor cells was enhanced by immunotherapy-activated CD8^+^ T cells. This increase of ferroptosis in tumor cells by CD8+ T cells contributes to the anti-tumor efficacy of immunotherapy [67]. Except for T cells, ferroptosis was also associated with the immune activity of macrophages, neutrophils, and B cells [68–70]. The current evidence suggests that the differentiation and activity of immune cells might be governed by lipid peroxidation, and immune cells might participate in regulating ferroptotic inflammation. Additionally, DAMPs released from ferroptotic cells could integrate with pattern recognition receptors (PRRs), such as toll-like receptor 4 (TLR4), which might mediate ferroptosis-related inflammatory responses [71]. Ferroptosis induced by bacterial infection might exacerbate the tissue injury in bacterial (e.g. *P. aeruginosa* and *M. tuberculosis*) pneumonia [72, 73]. Moreover, the ferroptotic macrophage might facilitate the spread of *M. tuberculosis*, which is detrimental to the host. Treatment with Fer-1 could significantly reduce bacterial load [73]. Therefore, ferroptosis has an interaction with immunity, and the activation of ferroptosis is associated with the development of bacterial infection-induced tissue injury. ARDS is also characterized by complex immunological changes, including uncontrolled inflammation and self-amplified injury [3, 74]. However, the ferroptosis-triggered lung and systematic immunological changes in ARDS remain unknown. Given the important role of ferroptosis in immunomodulation, we are looking forward to future studies which explore the role of ferroptosis-induced immunological changes in ARDS.

### Pyroptosis in ARDS

**Fig. 3:**
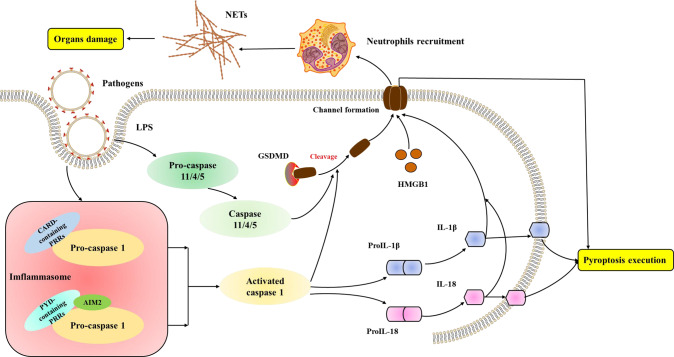
Molecular mechanisms of pyroptosis. The mechanisms of pyroptosis are divided into canonical pathways mediated by caspase 1 and noncanonical pathways activated by caspase 4/5/11. LPS lipopolysaccharide, CARD recruitment domain, PRRs pattern recognition receptors, PYD pyrin domain, AIM melanoma, IL interleukin, GSDMD gasdermin D, NETs neutrophil extracellular traps, HMGB1 high-mobility group box 1.

Pyroptosis is a type of cell death characterized by the formation of inflammasomes. Pyroptosis is defined as gasdermin D (GSDMD)-mediated regulated programmed necrotic cell death. GSDMD is a cytosolic protein that contains a specific cleavage site for inflammatory caspases (e.g., caspase 1, caspase 4, caspase 5, and caspase 11) [75–77]. The cleavage of GSDMD by activated caspase 1 results in the formation of a channel in the plasma membrane. Besides, inflammatory caspases cleave pro-IL-1β and pro-IL-18, converting them to the active form (IL-1β and IL-18) [78, 79]. The active form of these cytokines is then released through the channel or access the interstitial space upon pyroptosis execution, leading to the release of DAMPs and proinflammatory cytokines, which recruit the immune cells and amplify the inflammation. Therefore, Pyroptosis is considered as immunogenic cell death (Fig. 3).

Numerous studies have demonstrated that pyroptosis has an important role in ARDS. The cytokines induced by inflammasomes and caspases are critical mediators of ARDS. The pyroptosis of macrophages, endothelial cells, and neutrophils are involved in the development of ARDS. NOD‐like receptor protein 3 (NLRP3) inflammasome-mediated macrophage pyroptosis promotes high-mobility group box 1 (HMGB1) secretion [80]. HMGB1 further augments macrophage pyroptosis, amplifies inflammation, and aggravates ARDS [81]. Cellular pyroptosis is an important factor in the progression of COVID-19 to hypoxia and ARDS [82]. Inflammasomes activation releases extensive amounts of proinflammatory cytokines [83, 84]. Furthermore, GSDMD activation in monocytes or neutrophils by inflammasome contributes to coagulation, which may explain the high rate of venous and arterial thrombosis and the high mortality in severe COVID-19 patients [85]. Therefore, pyroptosis and tissue inflammation form a vicious cycle, ultimately leading to excessive inflammation and disease progression.

### Necroptosis in ARDS

**Fig. 4:**
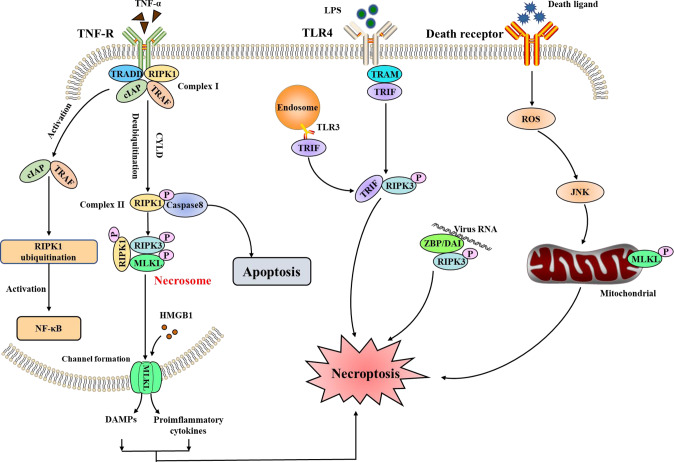
Molecular mechanisms of necroptosis. Necroptosis can be triggered by the receptors (e.g., TNFR1 and TLR3/4), which promote the assembly of the RIPK1–RIPK3–MLKL signaling complex. The ZBP1 can recognize the cytosolic DNA released from infecting microbes, which will activate RIPK3 and MLKL to lead to necroptosis. TNF tumor necrosis factor, R receptor, TRADD TNF-R-associated death domain, RIPK receptor-interacting kinase, cIAP cellular inhibitor of apoptosis protein, TRAF TNF-R-associated factors, CYLD cylindromatosis, MLKL mixed-lineage kinase domain-like pseudokinase, HMGB1 high-mobility group box 1, TLR toll-like receptor, TRIF TLR domain-containing adaptor-inducing interferon-β, ZBP/DAI Z-DNA-binding protein/DNA-dependent activator of interferon regulatory factors, JNK c-Jun N-terminal kinase, NF-κB nuclear factor kappa-B.

Necroptosis is a cell death program driven by the necrosome, resulting in necrotic morphology [86] (Fig. 4). The necrosome is composited by receptor-interacting kinase (RIPK) 1 and RIPK3. During the RIPK1-dependent necroptosis, activated RIPK1 executes autophosphorylation and interacts with RIPK3 via the RIP homotypic interaction motif (RHIM). Upon the formation of the necrosome, RIPK3 can phosphorylate mixed-lineage kinase domain-like pseudokinase (MLKL). Phosphorylated MLKL then translocates to cell membranes and forms a channel, increasing the permeabilization of the cell membrane [87]. Intracellular potents (e.g., HMGB1) are released through the channel and result in inflammation, accumulation of immune cells, and sustained immune response [88, 89]. Thus, necroptosis is also considered immunogenic cell death. Besides, toll-like receptor (TLR)3 and TLR4 activation can also promote the formation of the necrosomes and be involved in the progression of diseases (Fig. 4). Z-DNA-binding protein 1 (ZBP1) is another emerging innate immune sensor of viral Z-RNAs that recruits RIPK3 to activate necroptosis and apoptosis. Thus, the ZBP1 regulates the host defense responses during viral infection by initiating the programmed cell death pathways [90]. Furthermore, previous studies have pointed out that RIPK1 and RIPK3 also have an important role in apoptosis and pyroptosis [87, 91], making it difficult to identify the type of cell death in diseases.

Increased evidence has proven that necroptosis plays an important role in the pathogenesis of ARDS. Tamada et al. have demonstrated that necroptosis was the dominant type of cell death in alveolar epithelial in LPS-induced ARDS [92]. Bacterial infection can trigger necroptosis. For example, *Staphylococcus aureus* (*S. aureus*) and its toxins can efficiently induce necroptosis in polymorphonuclear leukocytes (PMN) or macrophages. The necroptosis of immune cells impedes bacterial clearance and increased pulmonary inflammation. The inhibition or deletion of RIPK3 and MLKL can improve bacterial pneumonia and prevent localized tissue damage [93, 94]. However, the role of necroptosis in virus-induced ARDS is highly controversial. SARS-CoV-2 infection can induce various inflammatory cytokines such as TNF-α and IFN-γ, which is beneficial for clearing the viral infection. Nevertheless, TNF-α and IFN-γ co-treatment can induce nitric oxide production and drive RIPK3/MLKL-mediated necroptosis, promoting tissue damage. Blocking the cytokine-mediated necroptosis may benefit COVID-19 patients [95]. Interestingly, in influenza, A virus (IAV) infection, the inhibition of necroptosis in mice failed to control IAV replication and led to lethal respiratory infection [96]. Thus, it is necessary to further identify the specific signaling axis in virus-induced necroptosis and determine the protective or detrimental effects of necroptosis in virus infection.

## The crosstalk among ferroptosis, pyroptosis, and necroptosis

Cell death plays a crucial role in combating infections and is implicated in various diseases. Different cell death pathways are involved in the development of a particular disease. For example, in renal ischemia-reperfusion injury, Zhao et al. have proven that ferroptosis, pyroptosis, and necroptosis are the predominant contributor to acute renal injury. Ferroptosis-related genes were mainly expressed in tubular epithelial cells, while the genes associated with pyroptosis and necroptosis were mainly expressed in macrophages [97]. Therefore, ferroptosis may exacerbate the damage. Inhibiting ferroptosis may be a more effective therapy compared to pyroptosis and necroptosis [98].

**Fig. 5:**
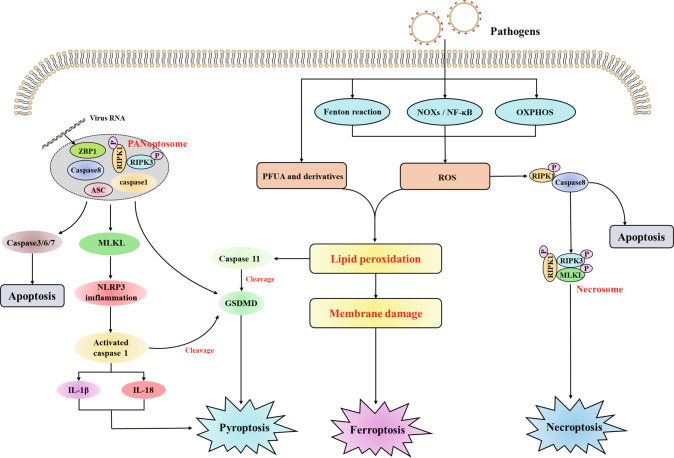
The crosstalk among pyroptosis, necroptosis, and ferroptosis. Diverse initiator and effector molecules involved in ferroptosis, pyroptosis, and necroptosis are interchangeable to promote cell death.

Increasing studies have identified the crosstalk of the different cell death pathways (Fig. 5). The interaction between ferroptosis, pyroptosis, and necroptosis is complex and they can operate in synergy to eliminate cells [99]. Several lines of evidence have proven the crosstalk between pyroptosis and necroptosis. Firstly demonstrated that RIPK3 was required for the NLRP3 inflammasome activity and the proIL-1β-associated ubiquitination was markedly increased in a RIPK3-dependent manner [100]. Several genetic experiments have also confirmed that the necroptosis signaling pathway can trigger the RIPK3–MLKL–NLRP3–Caspase-1 axis, leading to IL-1β maturation [101–103] (Fig. 5). Besides, apoptosis, pyroptosis, and necroptosis are interconnected by shared regulatory proteins and signaling pathways to consist of PANoptosis. The PANoptosome was initially shown to contain RIPK1, RIPK3, caspase 8, ASC, and caspase 1. The subsequent study showed that ZBP1 was also a component of PANoptosis, which recognizes the IAV. Then, these PANoptosome complexes promote the activation of downstream cell death receptors, representing apoptosis (caspase 3/6/7), pyroptosis (GSDMD), and necroptosis (MLKL). Thus, these findings highlight the important roles of different cell death pathways and their synergistic operation in eliminating cells which might promote the development of ARDS [104–106].

Oxidative stress is a critical mechanism contributing to PCDs. Kang et al. have demonstrated that lipid peroxidation can promote GSDMD-mediated pyroptosis in lethal polymicrobial sepsis. The conditional knockout of GPX4 has been shown to increase activation of the lipid peroxidation-dependent caspase 11 and GSDMD cleavage. Thus, GPX4 might negatively regulate pyroptosis by inhibiting lipid peroxidation, which prevents lethal polymicrobial sepsis in mice [68]. On the other hand, mitochondrial ROS is indispensable for the pathogenesis of ferroptosis and necroptosis. Mitochondrial ROS could promote autophosphorylation of the RIPK1 which recruits RIPK3, forming the functional necrosome (Fig. 5) [107]. The overexpression of GPX4 decreases the levels of mitochondrial ROS, thereby preventing ferroptosis and necroptosis [108]. In addition, knockout of ACSL4 can inhibit ferroptosis in ferroptosis-sensitive murine and human cells, but increase the susceptibility to necroptosis. These studies indicate that there is an interconnectivity between ferroptosis and necroptosis [109]. Moreover, the latest study has indicated that the release of DAMPs from the plasma membrane pore may be a common feature among ferroptosis, pyroptosis, and necroptosis [110]. The release of the DAMPs triggered by ferroptosis may promote pyroptosis and necroptosis. Thus, it is of interest to further explore the crosstalk among pyroptosis, necroptosis, and ferroptosis and clarify the interaction mechanisms in ARDS. Furthermore, it is also interesting to explore the specific DAMPs that can be targeted for preventing PCDs.

## Treatment of ARDS by targeting ferroptosis

As described above, ferroptosis plays a crucial role in the pathogenesis of ARDS, suggesting that there is great potential for the treatment of ARDS by targeting ferroptosis. Iron chelators and antioxidants would be effective treatments for ARDS. Iron chelators are used to remove the excess iron and inhibit the Fenton reaction, which reduces the ROS levels. Deferoxamine (DFO) is the first iron chelator approved by U.S. Food and Drug Administration (FDA) in 1968 [111]. Numerous studies have proven that DFO is effective in treating infection caused by bacteria, fungi, and viruses [112]. DFO can reduce the production of lipid peroxidants (e.g., MDA and 4-HNE) to prevent further damage. Thus, DFO has been hypothesized to have beneficial immunomodulatory and antiviral effects in defense against SARS-CoV-2 infection [113]. Further studies should be conducted to explore the therapeutic potential of DFO in COVID-19.

Oxidative stress is another critical mechanism to promote the accumulation of lipid peroxidants, which aggravates lung injury and contributes to the development of ARDS. Fer-1 is a specific inhibitor of ferroptosis that can ameliorate lipid peroxidation. In sepsis-induced ARDS, Fer-1 can improve inflammation and reduce the production of MDA and 4-HNE [8]. Besides, PUFA is an important source of lipid peroxidants. The inhibition of the metabolism of PUFA or knockdown of ACSL4 may be an effective therapy for ARDS [28, 114]. Antioxidants, such as vitamin E and vitamin C, can work synergistically to maintain redox balance and prevent lipid peroxidation. Furthermore, supplementation of exogenous GSH can also effectively ameliorate mitochondrial dysfunction [115]. N-acetylcysteine (NAC), a precursor of GSH, has been demonstrated to decrease the incidence and severity of influenza and influenza‐like illnesses [116]. NAC treatment attenuated pulmonary inflammation, pulmonary edema, MPO activity, and inflammatory cytokines (e.g., TNF-α, IL-6, and IL-1β) in mice with IAV-induced ARDS [117]. Interestingly, some studies have pointed out that NAC treatment can prevent the SARS-CoV-2 infection and restore the redox balance [118]. GSH has been proposed as a potential marker for the risk of development to severe COVID-19 and prevent oxidative damage when infection progresses [119]. Two COVID-19-associated ARDS patients were treated with GSH and showed significant improvement in dyspnea and reduction of cytokine storm syndrome [120]. Therefore, iron chelators and antioxidants may be effective therapies for ARDS patients. However, there is a lack of large-scale, high-quality randomized controlled trials of GSH for ARDS treatment. Future studies are needed to provide evidence for the effectiveness of GSH as a novel therapeutic approach for ARDS patients.

## Conclusion and perspectives

**Fig. 6:**
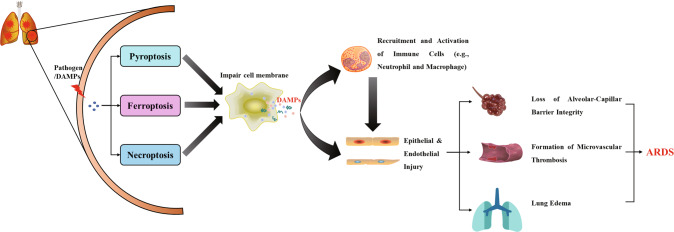
Ferroptosis, pyroptosis, and necroptosis are collaboratively involved in the development of ARDS. The cell death releasing the DAMPs (e.g., IL-1β and IL-18) that not only damage the epithelial cells and endothelial cells but also recruit and activate the immune cells to amplify the damage. Loss of epithelial–endothelial barrier integrity is associated with the development of microvascular thrombosis and lung edema.

The underlying regulatory mechanisms of ferroptosis and its link to diseases have been greatly explored since its discovery. Ferroptosis is a type of PCD characterized by the imbalance of iron metabolism and lipid metabolism. The accumulation of Fe^2+^ triggers the Fenton reaction to produce free radicals which damage the lipid membrane, resulting in lipid peroxidation. Besides, PUFAs can be oxidated by LPCAT3 and ACSL4 to generate the production of lipid ROS. Although we have understood the initiators, mediators, and regulators of ferroptosis, the ultimate executors of ferroptosis are still unclear [17]. Thus, the complex lipid metabolic network still needs to be further clarified. As described above, ferroptosis, pyroptosis, and necroptosis synergistically promote the development of ARDS (Fig. 6). The crosstalk among ferroptosis, pyroptosis, and necroptosis has received great attention. Lipid peroxidation not only induces ferroptosis but also leads to pyroptosis and necroptosis. Pore formation in the lipid membrane is an important mechanism for pyroptosis and necroptosis, but it is unknown if pore proteins also bind to the lipid membrane to induce ferroptosis. Furthermore, increased evidence demonstrated that there is mutual regulation among ferroptosis, pyroptosis, and necroptosis in diseases, but the regulatory mechanisms remain elusive and warrant further exploration.

Ferroptosis, as an immunogenic form of cell death, has been extensively studied in ARDS. However, the role of ferroptosis in virus-induced ARDS is yet to be determined. Compelling evidence has proven that ferroptosis inhibitors such as Fer-1 have positive effects in regulating inflammation and improving lung injury. Inhibition of ferroptosis can dramatically decrease the expression level of proinflammatory cytokines. Despite these findings connecting ferroptosis to the immune response in ARDS, the regulatory mechanisms remain unclear. The immunomodulatory effect of ferroptosis inhibitors in ARDS should also be further clarified. Despite the important role of ferroptosis in ARDS, specific markers to evaluate the ferroptosis-mediated immune response are still lacking. Lastly, the current research is limited to cell and animal studies, the clinical trials evaluating the effectiveness of ferroptosis inhibitors are still lacking. Given the important role of ferroptosis in ARDS, we really are looking forward to exploring the therapeutic potential of ferroptosis inhibitors in ARDS patients. Furthermore, due to the presence of multiple types of cell death in ARDS, combined interventions targeting multiple pathways may be a better therapy.

## References

[1] Ashbaugh DG, Bigelow DB, Petty TL, Levine BE (1967). Acute respiratory distress in adults. Lancet.

[2] Force ADT, Ranieri VM, Rubenfeld GD, Thompson BT, Ferguson ND, Caldwell E (2012). Acute respiratory distress syndrome: the Berlin Definition. JAMA.

[3] Matthay MA, Zemans RL, Zimmerman GA, Arabi YM, Beitler JR, Mercat A (2019). Acute respiratory distress syndrome. Nat Rev Dis Prim.

[4] Fan E, Brodie D, Slutsky AS (2018). Acute respiratory distress syndrome: advances in diagnosis and treatment. JAMA.

[5] Matthay MA, Zemans RL (2011). The acute respiratory distress syndrome: pathogenesis and treatment. Annu Rev Pathol.

[6] Thompson BT, Chambers RC, Liu KD (2017). Acute respiratory distress syndrome. N Engl J Med.

[7] Dixon SJ, Lemberg KM, Lamprecht MR, Skouta R, Zaitsev EM, Gleason CE (2012). Ferroptosis: an iron-dependent form of nonapoptotic cell death. Cell.

[8] Liu P, Feng Y, Li H, Chen X, Wang G, Xu S (2020). Ferrostatin-1 alleviates lipopolysaccharide-induced acute lung injury via inhibiting ferroptosis. Cell Mol Biol Lett.

[9] Li Y, Cao Y, Xiao J, Shang J, Tan Q, Ping F (2020). Inhibitor of apoptosis-stimulating protein of p53 inhibits ferroptosis and alleviates intestinal ischemia/reperfusion-induced acute lung injury. Cell Death Differ.

[10] Yang WS, Stockwell BR (2016). Ferroptosis: death by lipid peroxidation. Trends Cell Biol.

[11] Dolma S, Lessnick SL, Hahn WC, Stockwell BR (2003). Identification of genotype-selective antitumor agents using synthetic lethal chemical screening in engineered human tumor cells. Cancer Cell.

[12] Yang WS, Stockwell BR (2008). Synthetic lethal screening identifies compounds activating iron-dependent, nonapoptotic cell death in oncogenic-RAS-harboring cancer cells. Chem Biol.

[13] Wolpaw AJ, Shimada K, Skouta R, Welsch ME, Akavia UD, Pe’er D (2011). Modulatory profiling identifies mechanisms of small molecule-induced cell death. Proc Natl Acad Sci USA.

[14] Miotto G, Rossetto M, Di Paolo ML, Orian L, Venerando R, Roveri A (2020). Insight into the mechanism of ferroptosis inhibition by ferrostatin-1. Redox Biol.

[15] Ursini F, Maiorino M (2020). Lipid peroxidation and ferroptosis: The role of GSH and GPx4. Free Radic Biol Med.

[16] Xie Y, Hou W, Song X, Yu Y, Huang J, Sun X (2016). Ferroptosis: process and function. Cell Death Differ.

[17] Tang D, Kroemer G (2020). Ferroptosis. Curr Biol.

[18] Henricks PA, Nijkamp FP (2001). Reactive oxygen species as mediators in asthma. Pulm Pharm Ther.

[19] Zorov DB, Juhaszova M, Sollott SJ (2014). Mitochondrial reactive oxygen species (ROS) and ROS-induced ROS release. Physiol Rev.

[20] Doll S, Conrad M (2017). Iron and ferroptosis: a still ill-defined liaison. IUBMB Life.

[21] Yang WH, Huang Z, Wu J, Ding CC, Murphy SK, Chi JT (2020). A TAZ-ANGPTL4-NOX2 axis regulates ferroptotic cell death and chemoresistance in epithelial ovarian cancer. Mol Cancer Res.

[22] Shen Q, Liang M, Yang F, Deng YZ, Naqvi NI (2020). Ferroptosis contributes to developmental cell death in rice blast. N Phytol.

[23] Jourdain AA, Begg BE, Mick E, Shah H, Calvo SE, Skinner OS (2021). Loss of LUC7L2 and U1 snRNP subunits shifts energy metabolism from glycolysis to OXPHOS. Mol Cell.

[24] Stockwell BR, Friedmann Angeli JP, Bayir H, Bush AI, Conrad M, Dixon SJ (2017). Ferroptosis: a regulated cell death nexus linking metabolism, redox biology, and disease. Cell 38分.

[25] Dierge E, Debock E, Guilbaud C, Corbet C, Mignolet E, Mignard L (2021). Peroxidation of n-3 and n-6 polyunsaturated fatty acids in the acidic tumor environment leads to ferroptosis-mediated anticancer effects. Cell Metab.

[26] Magtanong L, Ko PJ, To M, Cao JY, Forcina GC, Tarangelo A (2019). Exogenous monounsaturated fatty acids promote a ferroptosis-resistant cell state. Cell Chem Biol.

[27] Li Z, Jiang H, Ding T, Lou C, Bui HH, Kuo MS (2015). Deficiency in lysophosphatidylcholine acyltransferase 3 reduces plasma levels of lipids by reducing lipid absorption in mice. Gastroenterology.

[28] Doll S, Proneth B, Tyurina YY, Panzilius E, Kobayashi S, Ingold I (2017). ACSL4 dictates ferroptosis sensitivity by shaping cellular lipid composition. Nat Chem Biol.

[29] Lee H, Zandkarimi F, Zhang Y, Meena JK, Kim J, Zhuang L (2020). Energy-stress-mediated AMPK activation inhibits ferroptosis. Nat Cell Biol.

[30] Song X, Zhu S, Chen P, Hou W, Wen Q, Liu J (2018). AMPK-mediated BECN1 phosphorylation promotes ferroptosis by directly blocking system Xc(-) activity. Curr Biol.

[31] Li C, Zhang Y, Liu J, Kang R, Klionsky DJ, Tang D (2021). Mitochondrial DNA stress triggers autophagy-dependent ferroptotic death. Autophagy.

[32] Shintoku R, Takigawa Y, Yamada K, Kubota C, Yoshimoto Y, Takeuchi T (2017). Lipoxygenase-mediated generation of lipid peroxides enhances ferroptosis induced by erastin and RSL3. Cancer Sci.

[33] Tseng HC, Lin CC, Wang CY, Yang CC, Hsiao LD, Yang CM (2018). Lysophosphatidylcholine induces cyclooxygenase-2-dependent IL-6 expression in human cardiac fibroblasts. Cell Mol Life Sci.

[34] Zou Y, Li H, Graham ET, Deik AA, Eaton JK, Wang W (2020). Cytochrome P450 oxidoreductase contributes to phospholipid peroxidation in ferroptosis. Nat Chem Biol.

[35] Yan B, Ai Y, Sun Q, Ma Y, Cao Y, Wang J (2021). Membrane damage during ferroptosis is caused by oxidation of phospholipids catalyzed by the oxidoreductases POR and CYB5R1. Mol Cell.

[36] Yang WS, SriRamaratnam R, Welsch ME, Shimada K, Skouta R, Viswanathan VS (2014). Regulation of ferroptotic cancer cell death by GPX4. Cell.

[37] Das UN (2019). Saturated fatty acids, MUFAs and PUFAs regulate ferroptosis. Cell Chem Biol.

[38] Zhang Z, Guo M, Li Y, Shen M, Kong D, Shao J (2020). RNA-binding protein ZFP36/TTP protects against ferroptosis by regulating autophagy signaling pathway in hepatic stellate cells. Autophagy.

[39] Dixon SJ, Patel DN, Welsch M, Skouta R, Lee ED, Hayano M (2014). Pharmacological inhibition of cystine-glutamate exchange induces endoplasmic reticulum stress and ferroptosis. Elife.

[40] Ingold I, Berndt C, Schmitt S, Doll S, Poschmann G, Buday K (2018). Selenium utilization by GPX4 is required to prevent hydroperoxide-induced ferroptosis. Cell.

[41] Shimada K, Skouta R, Kaplan A, Yang WS, Hayano M, Dixon SJ (2016). Global survey of cell death mechanisms reveals metabolic regulation of ferroptosis. Nat Chem Biol.

[42] Gaschler MM, Andia AA, Liu H, Csuka JM, Hurlocker B, Vaiana CA (2018). FINO2 initiates ferroptosis through GPX4 inactivation and iron oxidation. Nat Chem Biol.

[43] Moosmayer D, Hilpmann A, Hoffmann J, Schnirch L, Zimmermann K, Badock V (2021). Crystal structures of the selenoprotein glutathione peroxidase 4 in its apo form and in complex with the covalently bound inhibitor ML162. Acta Crystallogr D Struct Biol.

[44] Zhou L, Chen J, Li R, Wei L, Xiong H, Wang C (2021). Metal-polyphenol-network coated prussian blue nanoparticles for synergistic ferroptosis and apoptosis via triggered GPX4 inhibition and concurrent in situ bleomycin toxification. Small.

[45] Ding Y, Chen X, Liu C, Ge W, Wang Q, Hao X (2021). Identification of a small molecule as inducer of ferroptosis and apoptosis through ubiquitination of GPX4 in triple negative breast cancer cells. J Hematol Oncol.

[46] Chen C, Wang D, Yu Y, Zhao T, Min N, Wu Y (2021). Legumain promotes tubular ferroptosis by facilitating chaperone-mediated autophagy of GPX4 in AKI. Cell Death Dis.

[47] Canli O, Alankus YB, Grootjans S, Vegi N, Hultner L, Hoppe PS (2016). Glutathione peroxidase 4 prevents necroptosis in mouse erythroid precursors. Blood.

[48] Fan R, Sui J, Dong X, Jing B, Gao Z (2021). Wedelolactone alleviates acute pancreatitis and associated lung injury via GPX4 mediated suppression of pyroptosis and ferroptosis. Free Radic Biol Med.

[49] Loboda A, Damulewicz M, Pyza E, Jozkowicz A, Dulak J (2016). Role of Nrf2/HO-1 system in development, oxidative stress response and diseases: an evolutionarily conserved mechanism. Cell Mol Life Sci.

[50] Kerins MJ, Ooi A (2018). The roles of NRF2 in modulating cellular iron homeostasis. Antioxid Redox Signal.

[51] Dodson M, Castro-Portuguez R, Zhang DD (2019). NRF2 plays a critical role in mitigating lipid peroxidation and ferroptosis. Redox Biol.

[52] Anandhan A, Dodson M, Schmidlin CJ, Liu P, Zhang DD (2020). Breakdown of an ironclad defense system: the critical role of NRF2 in mediating ferroptosis. Cell Chem Biol.

[53] Sun X, Ou Z, Chen R, Niu X, Chen D, Kang R (2016). Activation of the p62-Keap1-NRF2 pathway protects against ferroptosis in hepatocellular carcinoma cells. Hepatology.

[54] Dong H, Qiang Z, Chai D, Peng J, Xia Y, Hu R (2020). Nrf2 inhibits ferroptosis and protects against acute lung injury due to intestinal ischemia reperfusion via regulating SLC7A11 and HO-1. Aging.

[55] Menon AV, Liu J, Tsai HP, Zeng L, Yang S, Asnani A (2022). Excess heme upregulates heme oxygenase 1 and promotes cardiac ferroptosis in mice with sickle cell disease. Blood.

[56] Liu Q, Gao Y, Ci X (2019). Role of Nrf2 and its activators in respiratory diseases. Oxid Med Cell Longev.

[57] Sun Y, Chen P, Zhai B, Zhang M, Xiang Y, Fang J (2020). The emerging role of ferroptosis in inflammation. Biomed Pharmacother.

[58] Linkermann A, Stockwell BR, Krautwald S, Anders HJ (2014). Regulated cell death and inflammation: an auto-amplification loop causes organ failure. Nat Rev Immunol.

[59] Wen Q, Liu J, Kang R, Zhou B, Tang D (2019). The release and activity of HMGB1 in ferroptosis. Biochem Biophys Res Commun.

[60] Li X, Zhuang X, Qiao T (2019). Role of ferroptosis in the process of acute radiation-induced lung injury in mice. Biochem Biophys Res Commun.

[61] Zhou H, Li F, Niu JY, Zhong WY, Tang MY, Lin D (2019). Ferroptosis was involved in the oleic acid-induced acute lung injury in mice. Sheng Li Xue Bao.

[62] Qiang Z, Dong H, Xia Y, Chai D, Hu R, Jiang H (2020). Nrf2 and STAT3 alleviates ferroptosis-mediated IIR-ALI by regulating SLC7A11. Oxid Med Cell Longev.

[63] Yoshida M, Minagawa S, Araya J, Sakamoto T, Hara H, Tsubouchi K (2019). Involvement of cigarette smoke-induced epithelial cell ferroptosis in COPD pathogenesis. Nat Commun.

[64] Zhang Y, Zheng L, Deng H, Feng D, Hu S, Zhu L (2022). Electroacupuncture alleviates LPS-induced ARDS through alpha7 nicotinic acetylcholine receptor-mediated inhibition of ferroptosis. Front Immunol.

[65] Li J, Li M, Li L, Ma J, Yao C, Yao S (2022). Hydrogen sulfide attenuates ferroptosis and stimulates autophagy by blocking mTOR signaling in sepsis-induced acute lung injury. Mol Immunol.

[66] Stockwell BR, Jiang X (2019). A physiological function for ferroptosis in tumor suppression by the immune system. Cell Metab.

[67] Wang W, Green M, Choi JE, Gijon M, Kennedy PD, Johnson JK (2019). CD8(+) T cells regulate tumour ferroptosis during cancer immunotherapy. Nature.

[68] Kang R, Zeng L, Zhu S, Xie Y, Liu J, Wen Q (2018). Lipid peroxidation drives gasdermin D-mediated pyroptosis in lethal polymicrobial sepsis. Cell Host Microbe.

[69] Yotsumoto S, Muroi Y, Chiba T, Ohmura R, Yoneyama M, Magarisawa M (2017). Hyperoxidation of ether-linked phospholipids accelerates neutrophil extracellular trap formation. Sci Rep.

[70] Muri J, Thut H, Bornkamm GW, Kopf M (2019). B1 and marginal zone B cells but not follicular B2 cells require Gpx4 to prevent lipid peroxidation and ferroptosis. Cell Rep.

[71] Loynes CA, Lee JA, Robertson AL, Steel MJ, Ellett F, Feng Y (2018). PGE2 production at sites of tissue injury promotes an anti-inflammatory neutrophil phenotype and determines the outcome of inflammation resolution in vivo. Sci Adv.

[72] Dar HH, Anthonymuthu TS, Ponomareva LA, Souryavong AB, Shurin GV, Kapralov AO (2021). A new thiol-independent mechanism of epithelial host defense against *Pseudomonas aeruginosa*: iNOS/NO(*) sabotage of theft-ferroptosis. Redox Biol.

[73] Amaral EP, Costa DL, Namasivayam S, Riteau N, Kamenyeva O, Mittereder L (2019). A major role for ferroptosis in *Mycobacterium tuberculosis*-induced cell death and tissue necrosis. J Exp Med.

[74] Meyer NJ, Gattinoni L, Calfee CS (2021). Acute respiratory distress syndrome. Lancet.

[75] Kayagaki N, Stowe IB, Lee BL, O’Rourke K, Anderson K, Warming S (2015). Caspase-11 cleaves gasdermin D for non-canonical inflammasome signalling. Nature.

[76] Wang K, Sun Q, Zhong X, Zeng M, Zeng H, Shi X (2020). Structural mechanism for GSDMD targeting by autoprocessed caspases in pyroptosis. Cell.

[77] Wang Y, Gao W, Shi X, Ding J, Liu W, He H (2017). Chemotherapy drugs induce pyroptosis through caspase-3 cleavage of a gasdermin. Nature.

[78] Dinarello CA (2009). Immunological and inflammatory functions of the interleukin-1 family. Annu Rev Immunol.

[79] Borthwick LA (2016). The IL-1 cytokine family and its role in inflammation and fibrosis in the lung. Semin Immunopathol.

[80] Hou L, Yang Z, Wang Z, Zhang X, Zhao Y, Yang H (2018). NLRP3/ASC-mediated alveolar macrophage pyroptosis enhances HMGB1 secretion in acute lung injury induced by cardiopulmonary bypass. Lab Investig.

[81] Qu L, Chen C, Chen Y, Li Y, Tang F, Huang H (2019). High-mobility Group Box 1 (HMGB1) and autophagy in acute lung injury (ALI): a review. Med Sci Monit.

[82] Vora SM, Lieberman J, Wu H (2021). Inflammasome activation at the crux of severe COVID-19. Nat Rev Immunol.

[83] Rodrigues TS, de Sa KSG, Ishimoto AY, Becerra A, Oliveira S, Almeida L (2021). Inflammasomes are activated in response to SARS-CoV-2 infection and are associated with COVID-19 severity in patients. J Exp Med.

[84] Junqueira C, Crespo A, Ranjbar S, Ingber J, Parry B, Ravid S, et al. SARS-CoV-2 infects blood monocytes to activate NLRP3 and AIM2 inflammasomes, pyroptosis and cytokine release. medRxiv [Preprint] 10.1101/2021.03.06.21252796. 2021.

[85] Wu C, Lu W, Zhang Y, Zhang G, Shi X, Hisada Y (2019). Inflammasome activation triggers blood clotting and host death through pyroptosis. Immunity.

[86] Linkermann A, Green DR (2014). Necroptosis. N. Engl J Med.

[87] Sauler M, Bazan IS, Lee PJ (2019). Cell death in the lung: the apoptosis-necroptosis axis. Annu Rev Physiol.

[88] Kaczmarek A, Vandenabeele P, Krysko DV (2013). Necroptosis: the release of damage-associated molecular patterns and its physiological relevance. Immunity.

[89] Yatim N, Cullen S, Albert ML (2017). Dying cells actively regulate adaptive immune responses. Nat Rev Immunol.

[90] Zhang T, Yin C, Boyd DF, Quarato G, Ingram JP, Shubina M (2020). Influenza virus Z-RNAs induce ZBP1-mediated necroptosis. Cell.

[91] Zhao H, Ning J, Lemaire A, Koumpa FS, Sun JJ, Fung A (2015). Necroptosis and parthanatos are involved in remote lung injury after receiving ischemic renal allografts in rats. Kidney Int.

[92] Tamada N, Tojo K, Yazawa T, Goto T (2020). Necrosis Rather than apoptosis is the dominant form of alveolar epithelial cell death in lipopolysaccharide-induced experimental acute respiratory distress syndrome model. Shock.

[93] Kitur K, Parker D, Nieto P, Ahn DS, Cohen TS, Chung S (2015). Toxin-induced necroptosis is a major mechanism of *Staphylococcus aureus* lung damage. PLoS Pathog.

[94] Greenlee-Wacker MC, Rigby KM, Kobayashi SD, Porter AR, DeLeo FR, Nauseef WM (2014). Phagocytosis of *Staphylococcus aureus* by human neutrophils prevents macrophage efferocytosis and induces programmed necrosis. J Immunol.

[95] Karki R, Sharma BR, Tuladhar S, Williams EP, Zalduondo L, Samir P (2021). Synergism of TNF-alpha and IFN-gamma triggers inflammatory cell death, tissue damage, and mortality in SARS-CoV-2 infection and cytokine shock syndromes. Cell.

[96] Kuriakose T, Man SM, Malireddi RK, Karki R, Kesavardhana S, Place DE (2016). ZBP1/DAI is an innate sensor of influenza virus triggering the NLRP3 inflammasome and programmed cell death pathways. Sci Immunol.

[97] Zhao Z, Wu J, Xu H, Zhou C, Han B, Zhu H (2020). XJB-5-131 inhibited ferroptosis in tubular epithelial cells after ischemia-reperfusion injury. Cell Death Dis.

[98] Martin-Sanchez D, Ruiz-Andres O, Poveda J, Carrasco S, Cannata-Ortiz P, Sanchez-Nino MD (2017). Ferroptosis, but not necroptosis, is important in nephrotoxic folic acid-induced AKI. J Am Soc Nephrol.

[99] Bedoui S, Herold MJ, Strasser A (2020). Emerging connectivity of programmed cell death pathways and its physiological implications. Nat Rev Mol Cell Biol.

[100] Duong BH, Onizawa M, Oses-Prieto JA, Advincula R, Burlingame A, Malynn BA (2015). A20 restricts ubiquitination of pro-interleukin-1beta protein complexes and suppresses NLRP3 inflammasome activity. Immunity.

[101] Lawlor KE, Khan N, Mildenhall A, Gerlic M, Croker BA, D’Cruz AA (2015). RIPK3 promotes cell death and NLRP3 inflammasome activation in the absence of MLKL. Nat Commun.

[102] Conos SA, Chen KW, De Nardo D, Hara H, Whitehead L, Nunez G (2017). Active MLKL triggers the NLRP3 inflammasome in a cell-intrinsic manner. Proc Natl Acad Sci USA.

[103] Gutierrez KD, Davis MA, Daniels BP, Olsen TM, Ralli-Jain P, Tait SW (2017). MLKL activation triggers NLRP3-mediated processing and release of IL-1beta independently of gasdermin-D. J Immunol.

[104] Zheng M, Karki R, Vogel P, Kanneganti TD (2020). Caspase-6 is a key regulator of innate immunity, inflammasome activation, and host defense. Cell.

[105] Lee S, Karki R, Wang Y, Nguyen LN, Kalathur RC, Kanneganti TD (2021). AIM2 forms a complex with pyrin and ZBP1 to drive PANoptosis and host defence. Nature.

[106] Messaoud-Nacer Y, Culerier E, Rose S, Maillet I, Rouxel N, Briault S (2022). STING agonist diABZI induces PANoptosis and DNA mediated acute respiratory distress syndrome (ARDS). Cell Death Dis.

[107] Zhang Y, Su SS, Zhao S, Yang Z, Zhong CQ, Chen X (2017). RIP1 autophosphorylation is promoted by mitochondrial ROS and is essential for RIP3 recruitment into necrosome. Nat Commun.

[108] Basit F, van Oppen LM, Schockel L, Bossenbroek HM (2017). van Emst-de Vries SE, Hermeling JC, et al. Mitochondrial complex I inhibition triggers a mitophagy-dependent ROS increase leading to necroptosis and ferroptosis in melanoma cells. Cell Death Dis.

[109] Muller T, Dewitz C, Schmitz J, Schroder AS, Brasen JH, Stockwell BR (2017). Necroptosis and ferroptosis are alternative cell death pathways that operate in acute kidney failure. Cell Mol Life Sci.

[110] Riegman M, Sagie L, Galed C, Levin T, Steinberg N, Dixon SJ (2020). Ferroptosis occurs through an osmotic mechanism and propagates independently of cell rupture. Nat Cell Biol.

[111] Mobarra N, Shanaki M, Ehteram H, Nasiri H, Sahmani M, Saeidi M (2016). A review on iron chelators in treatment of iron overload syndromes. Int J Hematol Oncol Stem Cell Res.

[112] Williams A, Meyer D (2009). Desferrioxamine as immunomodulatory agent during microorganism infection. Curr Pharm Des.

[113] Dalamaga M, Karampela I, Mantzoros CS (2020). Commentary: could iron chelators prove to be useful as an adjunct to COVID-19 treatment regimens?. Metabolism.

[114] Dixon SJ, Winter GE, Musavi LS, Lee ED, Snijder B, Rebsamen M (2015). Human haploid cell genetics reveals roles for lipid metabolism genes in nonapoptotic cell death. ACS Chem Biol.

[115] Aggarwal S, Dimitropoulou C, Lu Q, Black SM, Sharma S (2012). Glutathione supplementation attenuates lipopolysaccharide-induced mitochondrial dysfunction and apoptosis in a mouse model of acute lung injury. Front Physiol.

[116] De Flora S, Grassi C, Carati L (1997). Attenuation of influenza-like symptomatology and improvement of cell-mediated immunity with long-term N-acetylcysteine treatment. Eur Respir J.

[117] Zhang RH, Li CH, Wang CL, Xu MJ, Xu T, Wei D (2014). N-acetyl-l-cystine (NAC) protects against H9N2 swine influenza virus-induced acute lung injury. Int Immunopharmacol.

[118] Bartolini D, Stabile AM, Bastianelli S, Giustarini D, Pierucci S, Busti C (2021). SARS-CoV2 infection impairs the metabolism and redox function of cellular glutathione. Redox Biol.

[119] Kryukov EV, Ivanov AV, Karpov VO, Vasil’evich Alexandrin V, Dygai AM, Kruglova MP (2021). Association of low molecular weight plasma aminothiols with the severity of coronavirus disease 2019. Oxid Med Cell Longev.

[120] Horowitz RI, Freeman PR, Bruzzese J (2020). Efficacy of glutathione therapy in relieving dyspnea associated with COVID-19 pneumonia: a report of 2 cases. Respir Med Case Rep.

[121] Galluzzi L, Vitale I, Aaronson SA, Abrams JM, Adam D, Agostinis P (2018). Molecular mechanisms of cell death: recommendations of the Nomenclature Committee on Cell Death 2018. Cell Death Differ.

[122] Tonnus W, Meyer C, Paliege A, Belavgeni A, von Massenhausen A, Bornstein SR (2019). The pathological features of regulated necrosis. J Pathol.

[123] Hotchkiss RS, Strasser A, McDunn JE, Swanson PE (2009). Cell death. N Engl J Med.

[124] Tang D, Chen X, Kang R, Kroemer G (2021). Ferroptosis: molecular mechanisms and health implications. Cell Res.

[125] Zhou Y, Zhou H, Hua L, Hou C, Jia Q, Chen J (2021). Verification of ferroptosis and pyroptosis and identification of PTGS2 as the hub gene in human coronary artery atherosclerosis. Free Radic Biol Med.

